# Distinct patterns of ventricular fibrillation onset in primary electrical diseases: insights from a retrospective multicentre registry

**DOI:** 10.1093/europace/euaf209

**Published:** 2025-09-30

**Authors:** Elodie Surget, Wally Mansogo Ada, Alessandra Pia Porretta, Constance Beyler, Adrien Bloch, Isabelle Denjoy, Michel Haissaguerre, Fabrice Extramiana

**Affiliations:** Centre hospitalier universitaire Robert Debré, Université Paris Cité, 48 bvd Sérurier, 75019 Paris, France; Reference Center for Inherited Arrhythmic Syndromes, Centre hospitalier universitaire Bichat, APHP, Université Paris Cité, 46 rue Henri Huchard, Paris 75018, France; Centre hospitalier universitaire Robert Debré, Université Paris Cité, 48 bvd Sérurier, 75019 Paris, France; Centre hospitalier universitaire Robert Debré, Université Paris Cité, 48 bvd Sérurier, 75019 Paris, France; Service of Cardiology, Heart and Vessel Department, Centre Hospitalier Universitaire Vaudois, Lausanne, Switzerland; Centre hospitalier universitaire Robert Debré, Université Paris Cité, 48 bvd Sérurier, 75019 Paris, France; Institut de cardiologie, hôpital Pitié-Salpêtrière, Inserm UMRS 1166-ICAN (Institute of CardioMetabolism and Nutrition), AP-HP, Sorbonne université, ACTION Study Group, Paris 75013, France; Centre hospitalier universitaire Robert Debré, Université Paris Cité, 48 bvd Sérurier, 75019 Paris, France; Reference Center for Inherited Arrhythmic Syndromes, Centre hospitalier universitaire Bichat, APHP, Université Paris Cité, 46 rue Henri Huchard, Paris 75018, France; Cardiac Electrophysiology and Stimulation, Cardiology Department, Bordeaux University Hospital (CHU), Pessac, France; IHU Liryc, Electrophysiology and Heart Modeling Institute, Foundation Bordeaux Université, Bordeaux, France; Reference Center for Inherited Arrhythmic Syndromes, Centre hospitalier universitaire Bichat, APHP, Université Paris Cité, 46 rue Henri Huchard, Paris 75018, France

**Keywords:** Ventricular fibrillation, Chanellopathy, Idiopathic ventricular fibrillation, Long QT syndrome, Catecholaminergic polymorphic ventricular tachycardia, Brugada syndrome

## Abstract

**Aims:**

Ventricular fibrillation (VF) initiation is influenced by the underlying arrhythmia mechanism. Long QT syndrome (LQTS), catecholaminergic polymorphic ventricular tachycardia (CPVT), Brugada syndrome (BS), and idiopathic ventricular fibrillation (IVF) share overlapping clinical features that often hamper their diagnosis. This study aims to identify distinctive features of VF pattern that may provide additional diagnostic tools to discriminate them.

**Methods and results:**

We included consecutive patients affected by LQTS, CPVT, BS, or IVF with recurrent VF episodes. The evaluation of VF episodes documented on electrocardiogram or implantable cardioverter-defibrillator recordings allowed us to assess different features of VF episodes including sinus rhythm preceding VF, the trigger’s coupling interval (CI) initiating VF, and the mean cycle length of VF (VFCL) of the 10 first VF beats. Among 2399 patients with LQTS (*n* = 1587), CPVT (*n* = 155), BS (*n* = 584), or IVF (*n* = 62), 76 episodes of VF were identified in 30 patients. Catecholaminergic polymorphic ventricular tachycardia patients presented higher heart rate preceding VF (CL = 359 ± 26 ms; *P* < 0.001) and shorter trigger’s CI initiating VF (256 ± 21 ms; *P* < 0.001). Short-long-short sequence and very long CI (>450 ms) of trigger initiating VF were mostly observed in LQTS (54%; *P* < 0.001 and 68%, respectively). The VFCL was shorter in CPVT patients (168 ± 15 ms; *P* < 0.001). Based on these results, we created a discrimination algorithm to support the diagnostic process in clinical practice.

**Conclusion:**

Primary electrical diseases display distinct VF patterns. The comprehensive analysis of VF pattern, rather than of isolated VF features, holds then a major potential to support diagnostic accuracy and consequently refine therapeutic strategies in patients and in their relatives.

## Graphical Abstract

**Graphical Abstract:**
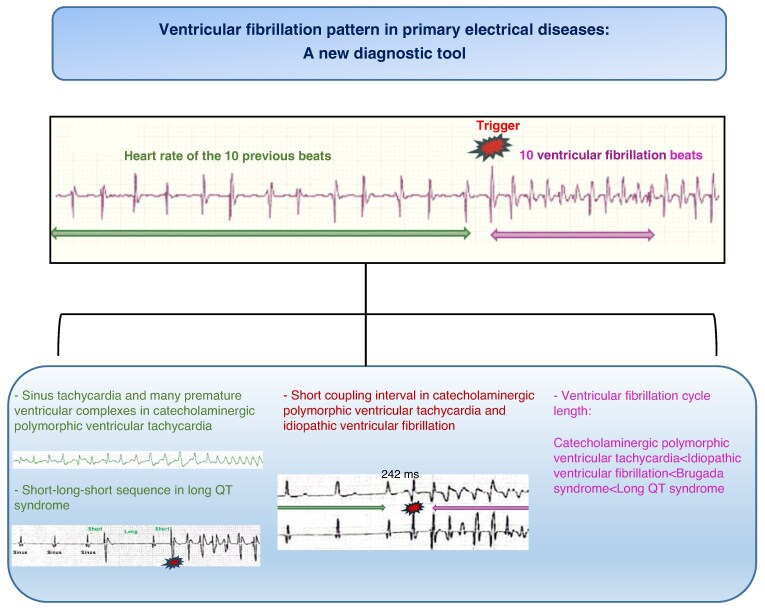

What’s new?

- This study provides the first comparative analysis of ventricular fibrillation (VF) initiation patterns across four primary electrical heart diseases: long QT syndrome (LQTS), catecholaminergic polymorphic ventricular tachycardia, Brugada syndrome, and idiopathic ventricular fibrillation (IVF).
- We identified disease-specific VF features, including distinct sinus rhythm preceding VF, trigger coupling intervals (CIs), and early VF cycle lengths (VFCLs).
- Catecholaminergic polymorphic ventricular tachycardia was associated with faster sinus rhythm and shorter VFCL, while LQTS presented with longer CIs and frequent short-long-short sequences.
- Idiopathic ventricular fibrillation episodes were most frequently initiated by short-coupled triggers (CI ≤ 320 ms), but overlap with other entities highlighted the limitations of using trigger CI alone for diagnosis.
- Based on these findings, we proposed a diagnostic algorithm integrating multiple VF features that may help differentiate channelopathies from IVF.
- This approach should be considered hypothesis generating and may provide supportive information in selected contexts, particularly in patients with unexplained VF, rather than serving as a broadly applicable diagnostic tool.

## Introduction

The principal mechanism of sudden cardiac death (SCD) is ventricular fibrillation (VF),^1,2^ which, among young individuals, is usually attributable to primary electrical heart diseases.^3^ The diagnostic of these electrical disorders—including Brugada syndrome (BS), long QT syndrome (LQTS), catecholaminergic polymorphic ventricular tachycardia (CPVT), and early repolarization syndrome (ERS)—is made on the basis of extensive diagnostic workup.^4,5^ Idiopathic VF (IVF), which is often initiated by short-coupled premature ventricular contractions (PVCs),^6–9^ is a diagnosis of exclusion and reserved to patients who survived a VF episode, without evidence for an underlying structural or electrical heart disease. However, recent studies demonstrated that a comprehensive diagnostic workup is carried out in only a small proportion of unexplained SCD (∼16%)^2^—leading to an overuse of the diagnosis of IVF.^10,11^ In addition, since some overlapping clinical features exist between the various entities—including borderline QT values^12^ or long coupled PVCs in IVF^13^—their clinical diagnosis still remains particularly challenging, even after a comprehensive workup. Yet, the correct diagnosis of such electrical disorders—which are in most cases inherited—is crucial for the optimization of specific therapies and the implementation of preventive strategies among relatives.

The aim of this study was to provide an adjunctive tool to support the diagnostic process in primary electrical diseases. As these entities have different electrophysiological characteristics, we speculated that they would display distinct mechanisms of VF. To address this hypothesis, we analysed their pattern of VF initiation, recorded on electrocardiogram (ECG) and implantable cardioverter-defibrillator (ICD) stored electrograms.

## Methods

### Study population

Consecutive patients with primary electrical disease from APHP University hospitals (Bichat and Debré, Paris) were retrospectively reviewed between 1996 and 2024. Patients with polymorphic ventricular tachycardia (PVT) or VF episodes (that we will group under the term of VF episodes) documented on ECG or ICD recordings were included. The diagnosis of the underlying cardiac disease was made on the basis of extensive diagnostic testing as per published guidelines.^5^ Structural heart diseases (SHDs) were ruled out using echocardiography, coronary angiography with ergonovine infusion when appropriate, and cardiac magnetic resonance imaging.

For patients without evidence of SHD, the following diagnosis criteria were used for primary electrical diseases:

- Brugada syndrome was diagnosed in patients who had a J-point elevation of ≥2 mV with coved ST elevation and T wave inversion in at least one right precordial ECG lead, V1 or V2, positioned in the second, third, or fourth intercostal spaces—occurring either spontaneously or after a provocative drug test with the intravenous administration of Class I antiarrhythmic drugs.
- Long QT syndrome was diagnosed in patients with a QTc of ≥480 ms (in repeated 12-lead ECGs or during 24-h Holter ECG or exercise testing) or a LQTS risk score of >3.
- Early repolarization syndrome was diagnosed in the presence of a J-point elevation of ≥1 mm in two or more contiguous inferior and/or lateral leads of a standard 12-lead ECG in a patient resuscitated from otherwise unexplained VF.
- Catecholaminergic polymorphic ventricular tachycardia was diagnosed in the presence of bidirectional PVCs or PVT during exercise testing or isoprenaline infusion (when exercise testing was not feasible).
- Idiopathic ventricular fibrillation diagnosis was reserved for patients who survived a VF episode without evidence for underlying structural or electrical heart diseases.

Patients with catheter ablation before the documented VF episode were excluded.

Genetic testing was performed by sequencing a set of 98 cardiomyopathy- and arrhythmia-related genes by using the HaloPlex capturing system (Agilent Technologies, Santa Clara, CA). The five-tier terminology system of the American College of Medical Genetics and Genomics (ACMG)^14^ was used for variant classification. Their clinical significance was assessed according to the integrated analysis proposed by ACMG recommendations. The study protocol complied with the ethical guidelines of the 1975 Declaration of Helsinki and its subsequent amendments. Patients were included in the MUTAVIT registry (Clinical Hospital Research Financing Program no. AOR04070 P040411), which was approved by the advisory committee for the protection of individuals in biomedical clinical research, Paris Saint-Louis.

### Ventricular fibrillation clinical characteristics

Clinical data including demographic characteristics, medical history, and circumstances of the VF event were collected. Electrical parameters—including heart rate, PR, QRS, and QTc intervals were collected on ECG performed shortly before or after VF episode. QTc interval was calculated using the Bazett formula (QTc = QT/√RR). The circumstances surrounding the VF episode were classified as occurrence during physical exertion, at rest or during sleep. Antiarrhythmic treatment at the time of VF episode was recorded.

### Characteristics of the ventricular fibrillation onset

We analysed VF episodes on ECG and ICD stored electrograms, when available. The ICD electrograms were reviewed to determine whether ICD therapies were delivered appropriately. Ventricular fibrillation was defined as a rapid, disorganized rhythm with variable cycle length (CL), morphology, and electrograms with low amplitude. Polymorphic ventricular tachycardia was diagnosed when electrograms displayed a ventricular tachycardia (VT) with a mostly constant amplitude but with variability in the morphology of the electrogram complexes. The CL measurements were not performed when the electrograms were too fragmented. Monomorphic VTs—defined as a VT with a uniform beat-to-beat CL and electrogram morphology—were excluded.

**Figure 1:**
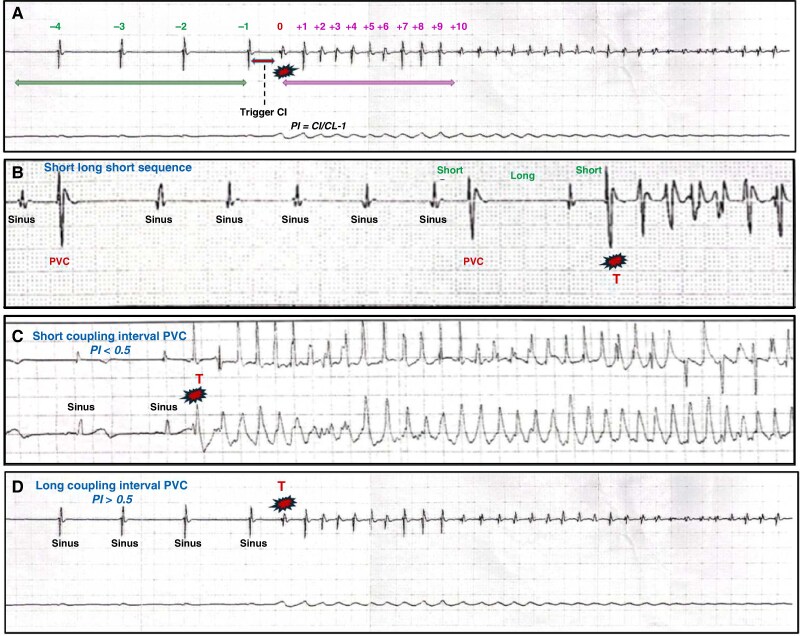
Analysis of the different sequences of VF episodes. (*A*) Nomenclature of the different sequences. Cycle A: the first beat preceding VF onset is labelled as ‘−1’ with the remaining preceding beats labelled in reverse chronological fashion to ‘−10’. Cycle B: the PVC-triggering VF episode is labelled as ‘0’. The following beats are labelled from ‘+1’ to ‘+10’. Cycle C: beats from ‘+11’ to ‘+20’. (*B*) Short coupling interval PVC. The prematurity of the trigger beat is assessed by determining the PI, which is defined as the coupling interval of the ectopic trigger normalized to the immediately preceding cycle length. A short coupling interval PVC is defined by a PI < 0.5. (*C*) Long coupling interval PVC. A long coupling interval PVC is defined by a PI > 0.5. (*D*) Short-long-short sequence. A short-long-short sequence is defined by a PVC, followed by a pause (CI > 1.2 × antecedent CI), and then by a short PVC. CI, coupling interval; CL, cycle length; PI, prematurity index; PVC, premature ventricular complex; T, trigger.

The analysis of the different sequences of VF episodes was performed as follows (*Figure 1A*):

#### The sinus rate preceding the onset of ventricular fibrillation

The first beat preceding VF onset was labelled as ‘−1’ with the remaining preceding beats labelled in reverse chronological fashion to ‘−10’. In this sequence, we analysed the presence of ≥1 isolated PVCs and the mean sinus rate (based on 3–10 consecutive beats). A sinus tachycardia was defined by a cardiac frequency of >100 b.p.m. The presence of a short-long-short (SLS) sequence—defined by a PVC with a disruption of the baseline rhythm, followed by a pause [coupling interval (CI) > 1.2 × antecedent CI], and then by a short PVC—was also specified^15^ (*Figure 1B*).

#### The trigger initiating ventricular fibrillation

The PVC-triggering VF episode was labelled as ‘0’. We analysed its CI and prematurity. The prematurity of the trigger beat was assessed by determining the prematurity index (PI), which was defined as the CI of the ectopic trigger normalized to the immediately preceding CL (PI = CI/CL − 1). A short-coupled PVC was defined by a PI < 0.5 and a late-coupled PVC by a PI ≥ 0.5^15, 16^ (*Figure 1C, D*).

#### The 10 first ventricular fibrillation cycle lengths

The 10 first consecutive VF beats were labelled from ‘+1’ to ‘+10’. V–V interval measurements were visually checked and measured manually in case of disagreement with the device sensing. The mean CL of VF (VFCL) was measured from beat ‘+1’ to ‘+10’.

### Statistical analysis

The dataset consisted of 76 VF episodes recorded in 30 patients. For each VF episode, we sought to determine the relationship between primary electrical disease type and the pattern of VF onset with (i) the sinus rate preceding the onset of VF, (ii) the CI and PI of the PVC initiating VF, and (iii) the 10 first VF CLs. Because each patient contributed one or more VF episodes to the dataset, each set of measurements cannot be considered independent. In addition, the number of VF episodes differed among subjects. To account for these factors, statistical analysis was performed using the generalized estimating equation (GEE) technique, which takes into account the varying number of observations that were obtained from each patient. Bivariate models initially were run to test the association between each predictor and each outcome separately. For these analyses, an exchangeable correlation was assumed, and robust variance estimation (R studio) was used to estimate the GEE models. Categorical variables were reported as numbers and percentages. Continuous variables were presented as mean ± standard error of mean (SEM) when normally distributed or median and inter-quantile ranges [p25; p75] when not normally distributed. A *P* < 0.05 was considered statistically significant. In order to discriminate primary electrical diseases, we created a contingency table based on the CI trigger, the cardiac frequency preceding the onset of VF, and the VFCL during the 10 first VF beats. Based on these findings, we developed a diagnostic algorithm integrating multiple VF features to differentiate channelopathies from IVF.

The CI cut-off values used in our VF discrimination algorithm were determined through ROC curve analysis. Specifically, we calculated the sensitivity and specificity of various CI thresholds and selected the cut-off values that maximized the Youden index (sensitivity + specificity—1). This method allowed us to identify thresholds that offer the best trade-off between sensitivity and specificity for clinical relevance.

## Results

### Patient characteristics

**Figure 2:**
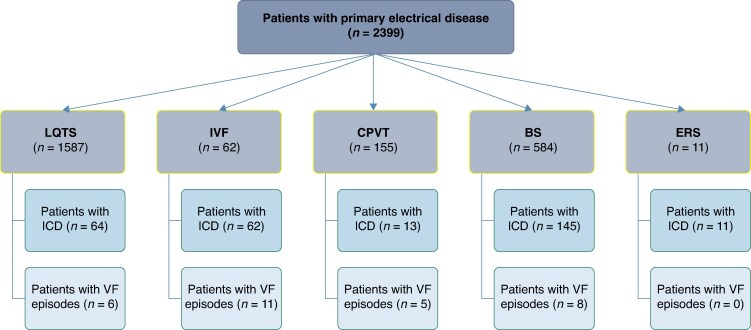
Flow chart. BS, Brugada syndrome; CPVT, catecholaminergic polymorphic ventricular tachycardia; ERS, early repolarization syndrome; IVF, idiopathic ventricular fibrillation; LQTS, long QT syndrome; SQT, short QT syndrome.

Out of 2399 patients with primary electrical disease, 30 met the inclusion criteria. Eight had BS, 6 LQTS, 5 CPVT, and 11 IVF (*Figure 2*). A total of 76 VF episodes were analysed: 3 on ECG and 73 on ICD stored electrograms. At the time of the VF episodes, 8 episodes occurred while the patient was being treated with beta-blockers, 10 with Class I antiarrhythmics (including 3 with flecainide for LQTS and CPVT and 7 with hydroquinidine for BS and IVF), and 7 with verapamil. One patient with CPVT had additional sympathectomy.

The baseline characteristics of the patients [mean age 34 ± 1 years; 16 (53%) women] with recorded VF episodes are summarized in *Table 1*. Clinically, male sex was more frequent in BS and less frequently represented among LQTS patients. Two patients had a family history of sudden cardiac arrest. Ninety-three per cent of patients had an ICD: two long QT patients did not have an ICD at the time of VF episode. Of these patients, 15/26 (58%) had an identified genetic mutation with *KCNQ1* in 2 and KCNH2 in 4 for LQTS, RYR2 in 4 and CASQ1 in 1 for CPVT, and SCN5A in 3 and ANK2 in 1 for BS.

**Table 1:** Population study

Number of patients	Total (*n* = 30)	LQTS (*n* = 6)	CPVT (*n* = 5)	IVF (*n* = 11)	BS (*n* = 8)
Female sex	16 (53)	6 (100)	4 (80)	6 (46)	0 (0)
Presence of ICD	28 (93)	4 (66)	5 (100)	11 (100)	8 (100)
Family history of SCD	0 (0)	0 (0)	0 (0)	0 (0)	2 (25)
Genetic testing	26/30 (87)	6/6 (100)	5/5 (100)	11 (100)	7/8 (87)
Gene variant found	15/26 (58)	6/6 (100)	5/5 (100)	0 (0)	4/7 (57)
**ECG parameters before VF**					
HR (b.p.m.)	64 ± 2	72 ± 2	52 ± 2	66 ± 2	75 ± 3
PR interval (ms)	176 ± 8	174 ± 1	156 ± 6	150 ± 2	221 ± 17
QRS duration during SR (ms)	104 ± 6	84 ± 12	96 ± 4	89 ± 4	135 ± 13
QTc duration (ms)	416 ± 10	452 ± 31	394 ± 5	393 ± 20	448 ± 20

Number of VF episodes	*n* = 76	*n* = 16	*n* = 11	*n* = 26	*n* = 23
**Antiarrhythmic treatment**					
No treatment	17 (31)	0 (0)	3 (33)	4 (17)	10 (53)
I	13 (24)	1 (33)	4 (45)	2 (9)	6 (31)
II	6 (11)	2 (67)	1 (11)	0 (0)	3 (16)
I + II	3 (6)	0 (0)	1(11)	2 (9)	0 (0)
III	0 (0)	0 (0)	0 (0)	0 (0)	0 (0)
I + IV	1 (2)	0 (0)	0 (0)	1 (4)	0 (0)
IV	14 (26)	0 (0)	0 (0)	14 (61)	0 (0)
Unknown	22	13	2	3	4

Values are presented as mean ± SEM or as *n* (%)

BS, Brugada syndrome; CPVT, catecholaminergic polymorphic ventricular tachycardia; ICD, implantable cardiac defibrillator; IVF, idiopathic ventricular fibrillation; LQTS, long QT syndrome; SCD, sudden cardiac death; SR, sinus rhythm; VF, ventricular fibrillation.

### Ventricular fibrillation clinical characteristics

Catecholaminergic polymorphic ventricular tachycardia patients experienced VF episode at a younger age compared to others (21 ± 1 years; *P* < 0.001). In 33 out of the 76 episodes, the circumstance of VF occurrence was unknown or not documented. Twenty-two episodes occurred at rest or during sleep and 21 during physical exertion (*Table 2*). For the cases with available data, episodes occurred more often at rest in IVF patients (87%; *P* < 0.001) and during physical exertion in CPVT ones (100%; *P* < 0.001). Episodes occurred during effort in 64% of LQTS.

**Table 2:** Ventricular fibrillation clinical characteristics

VF episodes	Total (*n* = 76)	LQTS (*n* = 16)	CPVT (*n* = 11)	IVF (*n* = 26)	BS (*n* = 23)	*P*
Age at VF episode (y)	34 ± 1	34 ± 2	21 ± 1	33 ± 2	40 ± 3	**<0.001**
**Circumstances of VF**						**<0.001**
Sleep or rest	22 (51)	5 (36)	0 (0)	13 (87)	4 (67)	
Effort	21 (49)	9 (64)	8 (100)	2 (13)	2 (33)	
Unknown	33	2	3	11	17	
**Time of day**						0.7
Day	45 (71)	7 (88)	5 (63)	16 (67)	17 (74)	
Night	18 (29)	1 (13)	3 (37)	8 (33)	6 (26)	
Unknown	13	8	3	2	0	

Values are presented as mean ± SEM or as *n* (%). The values shown in bold indicate statistically significant results (*P* < 0.05).

BS, Brugada syndrome; CPVT, catecholaminergic polymorphic ventricular tachycardia; LQTS, long QT syndrome; PVC, premature ventricular complex; VF, ventricular fibrillation.

### Ventricular fibrillation electrophysiological characteristics

**Figure 3:**
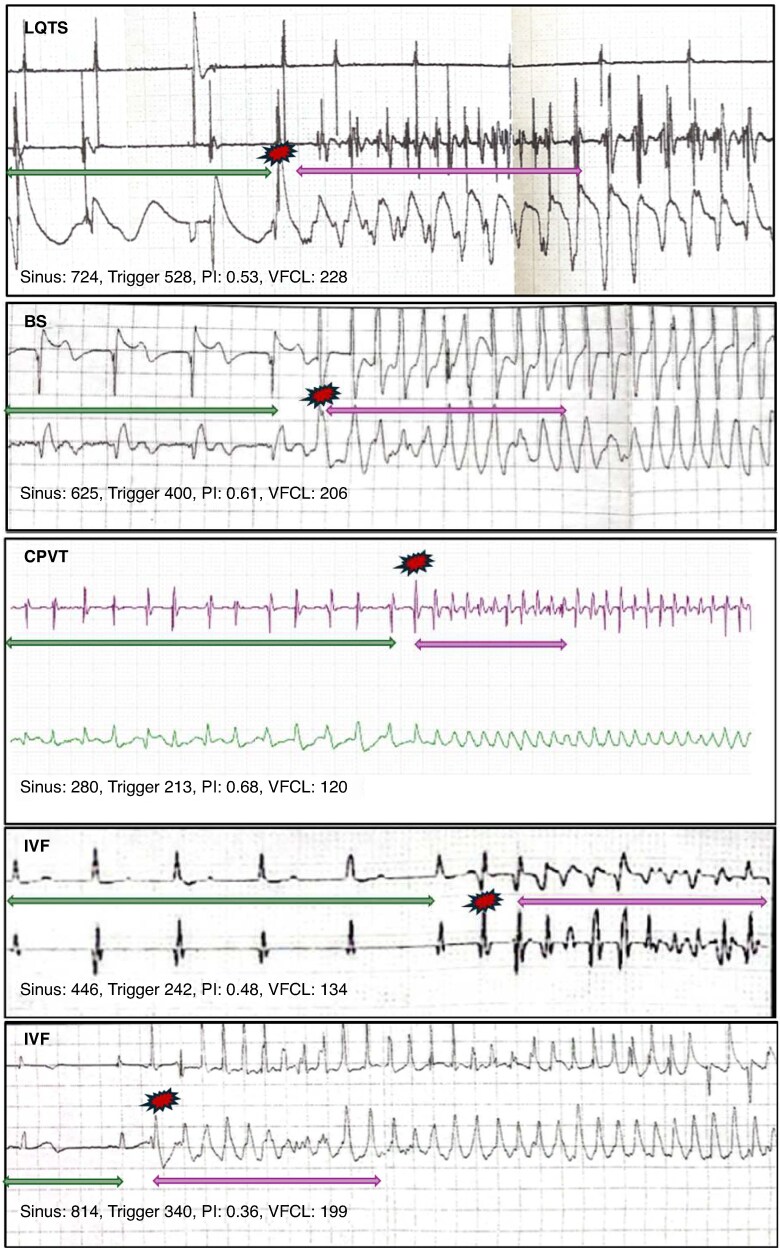
VF characteristics according to primary electrical disease. Stored intracardiac electrograms from patients with LQTS, BS, CPVT, and IVF. First panel: LQTS showed typically SLS sequence before VF onset. CI of trigger and VFCL are long. Second panel: VF in BS is induced by long-coupled trigger. VFCLs are also long (>200 ms). Third panel: CPVT patients presented sinus tachycardia before VF onset with short CI trigger and VFCL. Fourth panel: IVF mostly present with short CI trigger. Fifth panel: in some IVF cases, VF can be induced by long-coupled triggers. BS, Brugada syndrome; CPVT, catecholaminergic polymorphic ventricular tachycardia; IVF, idiopathic ventricular fibrillation; LQTS, long QT syndrome; PI, prematurity index.

The characteristics of the different sequences of VF episodes are shown in *Tables 3* and *4* and illustrated in *Figure 3*.

**Table 3:** Characteristics of the rhythm preceding VF

VF episodes	Total (*n* = 76)	LQTS (*n* = 16)	CPVT (*n* = 11)	IVF (*n* = 26)	BS (*n* = 23)	*P*
Sinus CL preceding VF	637 ± 24	806 ± 62	359 ± 26	644 ± 25	749 ± 27	**<0**.**001**
≥1 PVCs (*n* = 74)	47 (64)	12/14 (86)	11/11 (100)	12/26 (46)	12/23 (52)	**0**.**002**
SLS (*n* = 73)	13 (18)	7/13(54)	3/11 (27)	0/26 (0)	3/23 (13)	**<0**.**001**

Values are presented as mean ± SEM or as *n* (%). The values shown in bold indicate statistically significant results (*P* < 0.05).

BS, Brugada syndrome; CPVT, catecholaminergic polymorphic ventricular tachycardia; LQTS, long QT syndrome; PVC, premature ventricular complex; SLS sequence, short-long-short sequence; VF, ventricular fibrillation.

**Table 4:** Characteristics of the trigger and the 10 first beats of VF

VF episodes	Total (*n* = 76)	LQTS (*n* = 16)	CPVT (*n* = 11)	IVF (*n* = 26)	BS (*n* = 23)	*P*
Trigger CI (ms)	368 ± 14	513 ± 31	256 ± 21	302 ± 15	396 ± 13	**<0.001**
Trigger PI	0.52 ± 0.02	0.58 ± 0.05	0.73 ± 0.06	0.46 ± 0.01	0.53 ± 0.03	**<0.001**
Mean VFCL of the 10 first beats (ms)	206 ± 7	304 ± 14	168 ± 15	187 ± 4	208 ± 4	**<0.001**

Values are presented as mean ± SEM or as *n* (%). The values shown in bold indicate statistically significant results (*P* < 0.05).

BS, Brugada syndrome; CI, coupling interval; CPVT, catecholaminergic polymorphic ventricular tachycardia; ICD, implantable cardioverter defibrillator; LQTS, long QT syndrome; PI, prematurity index; PVC, premature ventricular complex; SLS sequence, short-long-short sequence; VF, ventricular fibrillation; VFCL, ventricular fibrillation cycle length.

#### The sinus rhythm preceding ventricular fibrillation

The heart rate preceding VF episode was higher in CPVT patients (CL = 359 ± 26 ms) than in others (CL = 644 ± 25 ms in IVF, 749 ± 27 ms in BS, and 806 ± 62 ms in LQTS; *P* < 0.001) (*Table 3*). Premature ventricular contractions—mainly with bigeminy—were present in all cases from CPVT patients, in 86% of LQTS, 52% of BS, and 46% of IVF patients. The SLS sequence was usually observed in LQTS (54%; *P* < 0.001), less frequently in CPVT and BS, and never in IVF-related episodes (*Table 3*).

#### The trigger initiating ventricular fibrillation

Catecholaminergic polymorphic ventricular tachycardia patients had triggers with shorter CI (256 ± 21 ms) compared to others (302 ± 15 ms in IVF, 396 ± 13 ms in BS, and 513 ± 31 ms in LQTS; *P* < 0.001). The PVC initiating VF was short coupled in 69% of IVF patients (PI = 0.46 ± 0.01; *P* < 0.001). Triggers in CPVT patients were not considered short coupled (PI = 0.73 ± 0.06) due to the tachycardia preceding VF onset (*Table 4*).

**Figure 4:**
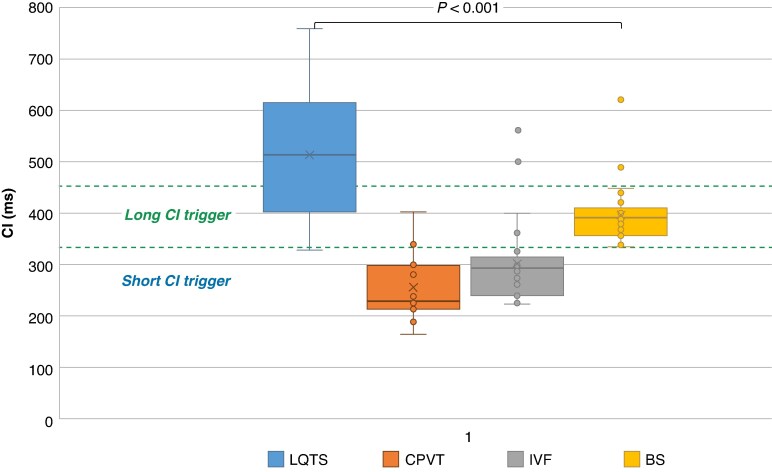
Distribution of trigger CI according to primary electrical disease. The box plots show that there is an overlap of trigger coupling interval between the different entities. Short CI triggers between 220 and 300 ms can be found in CPVT and IVF patients, whereas longer coupling intervals between 300 and 450 ms are present in IVF patients, Brugada, and LQT syndromes. BS, Brugada syndrome; CI, coupling interval; CPVT, catecholaminergic polymorphic ventricular tachycardia; LQTS, long QT syndrome; PI, prematurity index.

**Figure 5:**
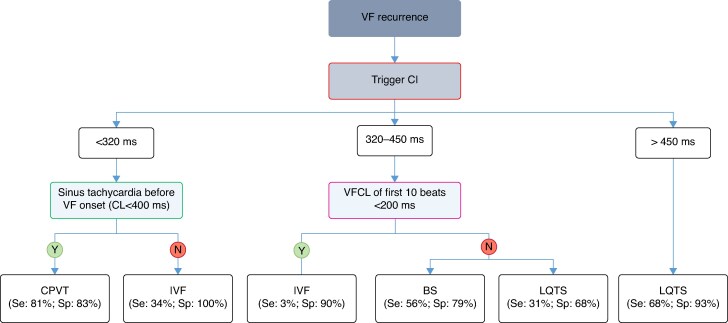
Algorithm for VF onset analysis to discriminate primary electrical diseases. For triggers with short CI (<320 ms), a sinus tachycardia before VF onset poses the diagnosis of CPVT. For trigger CIs between 320 and 450 ms, a VFCL of the first 10 beats < 200 ms is in favour of IVF. Very long CI > 450 ms is associated with LQTS. The percentage of diagnoses carried out is in brackets. BS, Brugada syndrome; CI, coupling interval; CL, cycle length; CPVT, catecholaminergic polymorphic ventricular tachycardia; IVF, idiopathic ventricular fibrillation; LQTS, long QT syndrome; N, no; PI, prematurity index; VF, ventricular fibrillation; VFCL, ventricular fibrillation cycle length; Y, yes.

The distribution of the trigger CIs is shown in *Figure 4*. To note, there was an overlap in trigger CI distribution between IVF and the other electrical disorders. Actually, 15% of IVF episodes were initiated by a trigger with a CI > 350 ms. As the value of trigger CI alone was not a discriminatory factor on its own, we established an algorithm (*Figure 5*) to discriminate IVF from LQTS and CPVT through the complete sequence analysis of VF episode. For triggers with short CI (200–320 ms), a sinus tachycardia (<400 ms) before VF onset posed the diagnosis of CPVT in 81% of cases. Instead, when trigger CI was between 320 and 450 ms, a VFCL of the first 10 beats of >200 ms usually eliminated the diagnosis of IVF (specificity = 90%). Very long CI > 450 ms were associated with LQTS in 68% of cases.

#### The 10 first mean cycle length of ventricular fibrillation

The mean VFCL was significantly shorter in CPVT patients (168 ± 15 ms) compared to others (187 ± 4 ms in IVF, 208 ± 4 ms in BS, and 304 ± 14 ms in LQTS; *P* < 0.001).

## Discussion

This study examined the characteristics of VF onset in primary electrical diseases. We demonstrated that each entity presented a specific VF pattern according to their electrophysiological properties, which allowed to discriminate them. For the first time, we established an algorithm that supports the diagnostic process of these entities and therefore refine therapeutic strategies in patients and their families.

### Idiopathic ventricular fibrillation and channelopathies

Idiopathic ventricular fibrillation is a diagnosis of exclusion, initially characterized by a short-coupled PVC (<320 ms) initiating VF.^6,8^ A subset of IVF is induced by short-coupled PVCs that arise from the Purkinje system in ∼90% of cases.^6,7^ Rarely, PVCs can arise from the right ventricular outflow tract,^17^ the papillary muscles,^18^ or the ventricular myocardium.^19^ This important heterogeneity of presentation makes its diagnosis often challenging. However, prior studies showed that IVF can also be triggered by PVCs with longer CIs, with an estimation of 17% of cases in a recent review.^7,20,21^ In our study, 4/26 (15%) of VF episodes were initiated by a PVC with a CI > 350 ms corroborating these previous results.^13,21^ Its association, in some cases, with microstructural myocardial alterations,^22^ explains also this heterogeneity of CIs. Indeed, Haïssaguerre *et al.*^23^ demonstrated that two subtypes of IVF could be distinguished: one characterized by Purkinje ectopies and structurally normal myocardium and the other associated with microstructural myocardial alterations. For this latter, the CIs of triggers were significantly longer. In our study, the shorter CI values in IVF overlapped with those observed in CPVT, and the longer ones with those found in LQTS. Note that, in our study, the CI values in CPVT were mostly <320 ms, whereas prior studies^24,25^ have generally established it to be around 400 ms. This discrepancy may stem from differences in methodology, as prior data on PVC CIs in CPVT were typically derived from exercise testing rather than spontaneous VF events. Our findings suggest that VF-triggering PVCs in CPVT may occur earlier than previously recognized, particularly under conditions of high adrenergic tone.

The apparent homogeneity of VF initiation parameters within each disease group (notably the narrow standard deviation for PVC CI and VFCL) is interesting. This may reflect the underlying electrophysiological properties specific to each condition rather than a random statistical finding. Nevertheless, these findings should be interpreted cautiously given the limited sample size and retrospective nature of the study. Therefore, the value of trigger CI alone was not a sufficient discriminatory factor on its own. As illustrated in *Figure 5*, additional parameters as SR preceding VF, SLS sequence, and VFCL added independent information to refine discrimination between these entities.

### Ventricular fibrillation characteristics

Ventricular fibrillation pattern is influenced by the autonomic nervous system and the electrophysiological properties (conduction and refractoriness) of cardiac tissue, which both determine the mechanisms of VF initiation and maintenance. Several studies^26,27^ have already analysed the pattern of VF initiation in different SHDs to gain insight into their arrhythmia mechanism and to optimize device programming. Usually, SHD-related VF presents a sinus tachycardia before VF onset^28,29^ with a long-coupled PVC initiating VF—especially in patients with impaired ventricular function and non-ischaemic dilated cardiomyopathy.^15^ According to previous studies, SLS sequences were rarely observed (<15%),^15,30^ and the mean VFCL was reported to be between 220 and 250 ms.^27^ Conversely, only few studies described VF onset in channelopathies.

In our study, all CPVT patients presented episodes during physical activity. The sequence before VF onset was characterized by numerous PVCs with a major tachycardia (359 ± 26 ms)—indicating a high sympathetic tone. This result indicates a proarrhythmic role for sympathetic activity in CPVT patients (by leading to delayed after depolarizations via premature sarcoplasmic reticulum calcium release mediated by β adrenergic receptor activation^31,32^). Conversely, the SLS sequences (which increase heterogeneity of repolarization necessary to induce PVCs via early after depolarization mechanism^33^) were typically found in LQTS (*P* < 0.001) Finally, the VFCL, which is mainly determined by conduction and repolarization properties of the cardiac tissue, was shorter in CPVT patients (*P* < 0.001) compared to IVF, BS, and LQTS ones.

### Clinical practical implications

In this study, we identified distinctive features of VF pattern in channelopathies and IVF that allowed us to establish an algorithm to better discriminate them. While this algorithm is not intended to replace a comprehensive diagnostic evaluation, it may be useful as an adjunctive tool in selected cases, particularly when conventional investigations are inconclusive or non-diagnostic. This is especially relevant given the limited diagnostic yield of standard tests in channelopathies, where genotype-negative or borderline cases remain frequent.

As the complete diagnostic workup to diagnose IVF is rarely carried out, the use of additional diagnostic criteria, such as this algorithm, can correct a misdiagnosis of IVF and allow the initiation of specific therapies (such as the introduction of β-blockers) in CPVT and LQT patients, for example. This information could also help tailor management in specific scenarios: for instance, the initiation of β-blockers in patients with CPVT or LQTS, the avoidance of contraindicated drugs in LQTS and BS, and the optimization of ICD programming to prevent bradycardia in BS and SLS sequences in LQTS patients.

Nevertheless, we recognize that the clinical applicability of this algorithm is currently limited to specific contexts. Its use is restricted to patients with documented VF episodes (ICD, telemetry, and Holter), and its real-world value should therefore be considered as hypothesis generating rather than as a broadly applicable diagnostic tool. Finally, as CPVT, BS, and LQTS are often inherited, their diagnosis in the proband is also crucial for the screening and the implementation of preventive strategies among relatives.

### Limitations

First, due to the tachycardia and the high proportion of PVCs preceding VF onset in CPVT, the real beginning of VF was difficult to determine. Secondly, the number of VF events was small, and some patients were on Class I or III antiarrhythmics, which may influence the electrophysiological properties of the substrate and PVC CIs. Thirdly, while a majority of cases was analysed from ICD stored electrograms, three VF episodes were recorded on ECG that allowed less accurate measurements. Moreover, although our algorithm demonstrated internal consistency, its real-world applicability is limited by the fact that it relies on the availability of VF recordings, which are typically only present in patients with ICDs or monitored settings. Finally, no external validation of our proposed VF initiation algorithm was performed due to the rarity of documented VF events in patients with primary electrical diseases. This limitation reflects the very low incidence of spontaneous VF recordings in inherited arrhythmia syndromes: only 76 VF episodes were captured among 2399 patients in our registry. While this precluded validation in an independent cohort, future multicentre collaborative efforts will be required to validate this diagnostic algorithm in a larger, prospective setting.

## Conclusions

Our study demonstrates that IVF and channelopathies display distinct mechanisms of VF initiation and maintenance. These findings should be considered hypothesis generating and may provide supportive information in selected contexts, particularly in cases of diagnostic uncertainty. Comprehensive analysis of the VF pattern, rather than relying solely on the trigger CI, could offer additional diagnostic insight to correct potential misdiagnoses and guide appropriate therapeutic measures, lifestyle counselling, and family screening. However, broader applicability of this algorithm remains to be demonstrated in larger prospective cohorts.

## Data Availability

The data underlying this article cannot be shared publicly due to patient privacy and ethical restrictions. The anonymized data will be made available to qualified researchers upon reasonable request to the corresponding author.
