# Correction to: Distinct patterns of ventricular fibrillation onset in primary electrical diseases: insights from a retrospective multicentre registry

**DOI:** 10.1093/europace/euaf259

**Published:** 2025-10-14

This is a correction to: Elodie Surget, Wally Mansogo Ada, Alessandra Pia Porretta, Constance Beyler, Adrien Bloch, Isabelle Denjoy, Michel Haissaguerre, Fabrice Extramiana, Distinct patterns of ventricular fibrillation onset in primary electrical diseases: insights from a retrospective multicentre registry, *EP Europace*, Volume 27, Issue 9, September 2025, https://doi.org/10.1093/europace/euaf209

The following changes have been made to the originally published manuscript. The name of the second author has been emended to read: “Wally Mansogo Ada”.

